# Automatic classification of distal radius fracture using a two-stage ensemble deep learning framework

**DOI:** 10.1007/s13246-023-01261-4

**Published:** 2023-04-27

**Authors:** Hang Min, Yousef Rabi, Ashish Wadhawan, Pierrick Bourgeat, Jason Dowling, Jordy White, Ayden Tchernegovski, Blake Formanek, Michael Schuetz, Gary Mitchell, Frances Williamson, Craig Hacking, Kevin Tetsworth, Beat Schmutz

**Affiliations:** 1 CSIRO Australian e-Health Research Centre, Herston, QLD Australia; 2 Ingham Institute for Applied Medical Research, Sydney, NSW Australia; 3 South Western Clinical School, University of New South Wales, Sydney, Australia; 4 School of Mechanical, Medical and Process Engineering, Faculty of Engineering, Queensland University of Technology, Brisbane, QLD Australia; 5 Royal Brisbane and Women’s Hospital, Herston, QLD Australia; 6 Centre for Medical Radiation Physics, University of Wollongong, Wollongong, NSW Australia; 7 Institute of Medical Physics, The University of Sydney, Sydney, NSW Australia; 8 School of Mathematical and Physical Sciences, University of Newcastle, Newcastle, NSW Australia; 9 Medical School, University of Queensland, Brisbane, QLD Australia; 10 Jamieson Trauma Institute, Herston, QLD Australia; 11 Monash Medical Centre, Clayton, VIC Australia; 12 Ochsner Clinical School, University of Queensland School of Medicine, Brisbane, QLD Australia; 13 ARC Training Centre for Multiscale 3D Imaging, Modelling, and Manufacturing, Queensland University of Technology, Brisbane, QLD Australia; 14 Centre of Biomedical Technologies, Queensland University of Technology, Kelvin Grove, QLD Australia

**Keywords:** Deep learning, Ensemble learning, Distal radius fracture, X-ray

## Abstract

Distal radius fractures (DRFs) are one of the most common types of wrist fracture and can be subdivided into intra- and extra-articular fractures. Compared with extra-articular DRFs which spare the joint surface, intra-articular DRFs extend to the articular surface and can be more difficult to treat. Identification of articular involvement can provide valuable information about the characteristics of fracture patterns. In this study, a two-stage ensemble deep learning framework was proposed to differentiate intra- and extra-articular DRFs automatically on posteroanterior (PA) view wrist X-rays. The framework firstly detects the distal radius region of interest (ROI) using an ensemble model of YOLOv5 networks, which imitates the clinicians’ search pattern of zooming in on relevant regions to assess abnormalities. Secondly, an ensemble model of EfficientNet-B3 networks classifies the fractures in the detected ROIs into intra- and extra-articular. The framework achieved an area under the receiver operating characteristic curve of 0.82, an accuracy of 0.81, a true positive rate of 0.83 and a false positive rate of 0.27 (specificity of 0.73) for differentiating intra- from extra-articular DRFs. This study has demonstrated the potential in automatic DRF characterization using deep learning on clinically acquired wrist radiographs and can serve as a baseline for further research in incorporating multi-view information for fracture classification.

## Introduction

Distal radius fractures (DRFs) are one of the most common fractures treated in orthopaedic practice [1]. Projection radiography is the standard imaging modality used in the initial imaging examination of DRFs [2], including posteroanterior (PA) and lateral views. In some cases, an oblique view may also be acquired [3]. During the interpretation of a DRF radiograph, a description highlighting the characteristics of the fracture can be beneficial for guiding treatment options. Several fracture classification systems, such as the AO/OTA (Arbeitsgemeinschaft für Osteosynthesefragen / Orthopaedic Trauma Association) [4], Frykman [5] and the Older [6] systems, have been proposed to describe DRFs. However, these classification systems are known to have high inter- and intra-observer variance and poor reproducibility, which hinders the clinical reliability of these systems. Currently, there is still no universally accepted standard for DRF classification [3], however, a simple differentiation between intra- and extra-articular fractures remains useful for treatment guidance [1]. In extra-articular fractures, the fracture line spares the articular surface, while the intra-articular fractures extend into the articular surface [4]. Compared with extra-articular fractures, intra-articular fractures can be associated with articular cartilage injury which can have long term functional impacts on the wrist joint. They are generally more difficult to treat and may require CT scans for further evaluation prior to surgical management [7]. Although there are various ways to categorize DRFs, radiographic classification remains challenging, due to the extreme variability and subtlety of fracture patterns, and overlap of surrounding bones [8, 9].

In recent years, deep learning (DL) has made remarkable progress in medical image analysis to assist clinical decision making. However, developing robust machine learning frameworks for fracture classification on wrist X-ray is challenging due to: (1) clinically acquired wrist X-rays vary greatly in image quality, positioning and field of view (FOV); and (2) the wrist has a very complex surrounding anatomy and can have a wide range of injuries from minor to complex traumas [10]. There have only been a few attempts to classify DRF using machine learning. Tobler et al. [11] proposed to use the ResNet-18 [12] to classify DRFs into fragment displacement, joint involvement and multi-fragmental on fixed-size centre-cropped X-rays, however, this centre-cropping pre-processing may not always be applicable to other clinical data. Yang et al. [13] proposed a DRF AO type classification method based on the fusion of traditional texture feature extraction and DL-based feature extraction. As this approach extracts the two types of features on the whole X-ray image instead of the region of interest, the effectiveness of these descriptors can be affected if the image data vary greatly in FOV and artefacts. Currently, there is still a lack of evidence to support DRF classification using DL.

This study explores the feasibility of classifying intra- and extra-articular DRFs on wrist radiographs using a two-stage ensemble DL framework. In the first stage, the distal radius regions of interest (ROIs) were automatically localized by an ensemble model of YOLOv5 networks [14]. In the second stage, the DRFs in the detected ROIs were classified by an ensemble model of EfficientNet [15] as intra- or extra-articular. To deal with the class imbalance between intra- and extra-articular DRFs, the minimum distance approach [16] was used to select a suitable probability threshold to achieve a balanced classification sensitivity and specificity for the unseen testing data. The Gradient-weighted Class Activation Mapping (Grad-CAM) [17] was also used to highlight the discriminative area to provide visual explanation. The main contributions of this study are summarized as follows:

- A two-stage ensemble DL framework was proposed to classify DRFs as intra- and extra-articular fractures which have different patterns of fragmentation and may require different treatments.
- The distal radius ROI was firstly localized prior to the classification stage, which allows the framework to focus on the relevant anatomic region and cope with the variation of FOV in wrist radiographs.
- Ensemble learning strategy was adopted to alleviate the impact of data variance and limited size of the clinical dataset.
- The recent release of YOLO (You Only Look Once) object detection network [18], YOLOv5, and EfficientNet classification network were used in the ensembles.
- Evaluated on a clinically acquired wrist X-ray dataset, the proposed DL framework demonstrated effectiveness in differentiating intra- from extra-articular DRFs.

## Materials and methods

### Wrist X-ray data

**Fig. 1:**
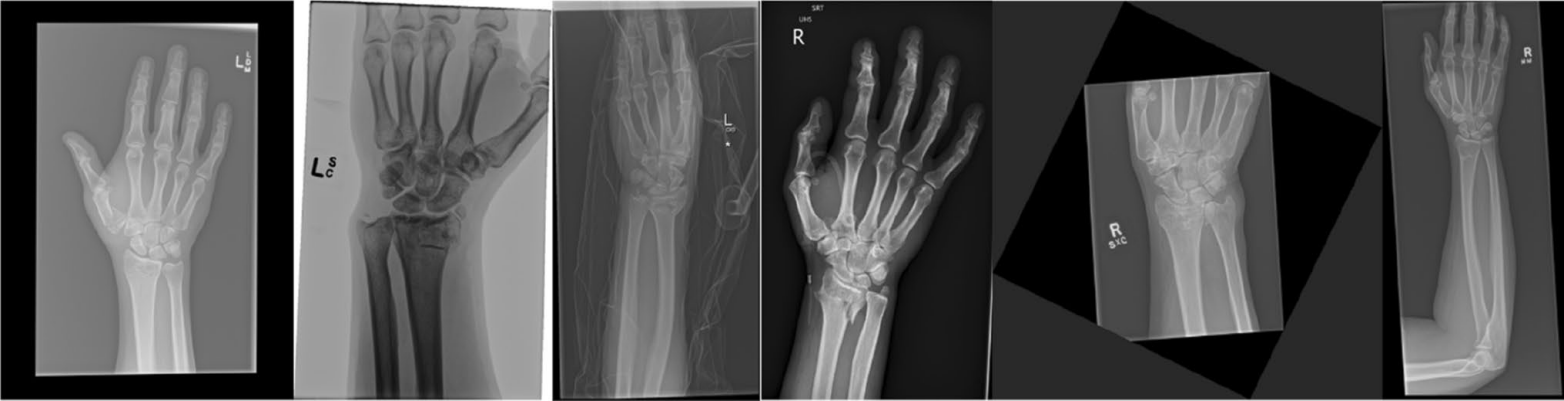
X-ray image examples from the dataset used in this study

Table 1 summarizes the statistics of the wrist X-ray dataset used in this study. Ethics approval for the data was granted by the Royal Brisbane and Women’s Hospital Human Research Ethics Committee (EC00172). Each study participant case contains a PA view and lateral view radiograph. Some cases also contain a PA view of the distal forearm (with the distal radius present) or a PA oblique view. Similar to Gan et al. [19], only the PA views were used in this study. The distal radius ROI ground truth which covers the articular surface and the DRF, was manually annotated by placing a bounding box over the relevant area and reviewed by a medical student. Each DRF was classified by three orthopaedic training registrars independently into extra- and intra-articular (partial-articular and complete articular). If there was a disagreement between the registrars, an orthopaedic consultant would further review the classification. The classification ground truth was decided by a majority voting across all clinician labels. If there was a tie between the class labels, the case would not be used. The participant may have both left and right wrists fractured, and the fractured wrist could be present in more than one image (e.g., a DRF is present in both wrist and forearm PA view X-rays). Thus, the dataset used in this study contains 400 X-rays of 349 DRFs (260 intra- and 89 extra-articular) from 340 patients. The dataset was randomly partitioned into 284 cases for training and 56 cases for testing. The X-ray images range from 0.097 to 0.168 mm in pixel spacing, 812 to 3713 pixels in height and 592 to 3648 in width. Figure 1 visualizes several image examples from the dataset, which vary greatly in contrast, FOV and artefacts.

**Table 1:** Summary statistics for the dataset used in this study

	Total			Train/validation			Test		
No. cases	340			284			56		
No. fractures	349	Intra	260	292	Intra	218	57	Intra	42
		Extra	89		Extra	74		Extra	15
No. images	400			334			66

### DRF classification framework

**Fig. 2:**
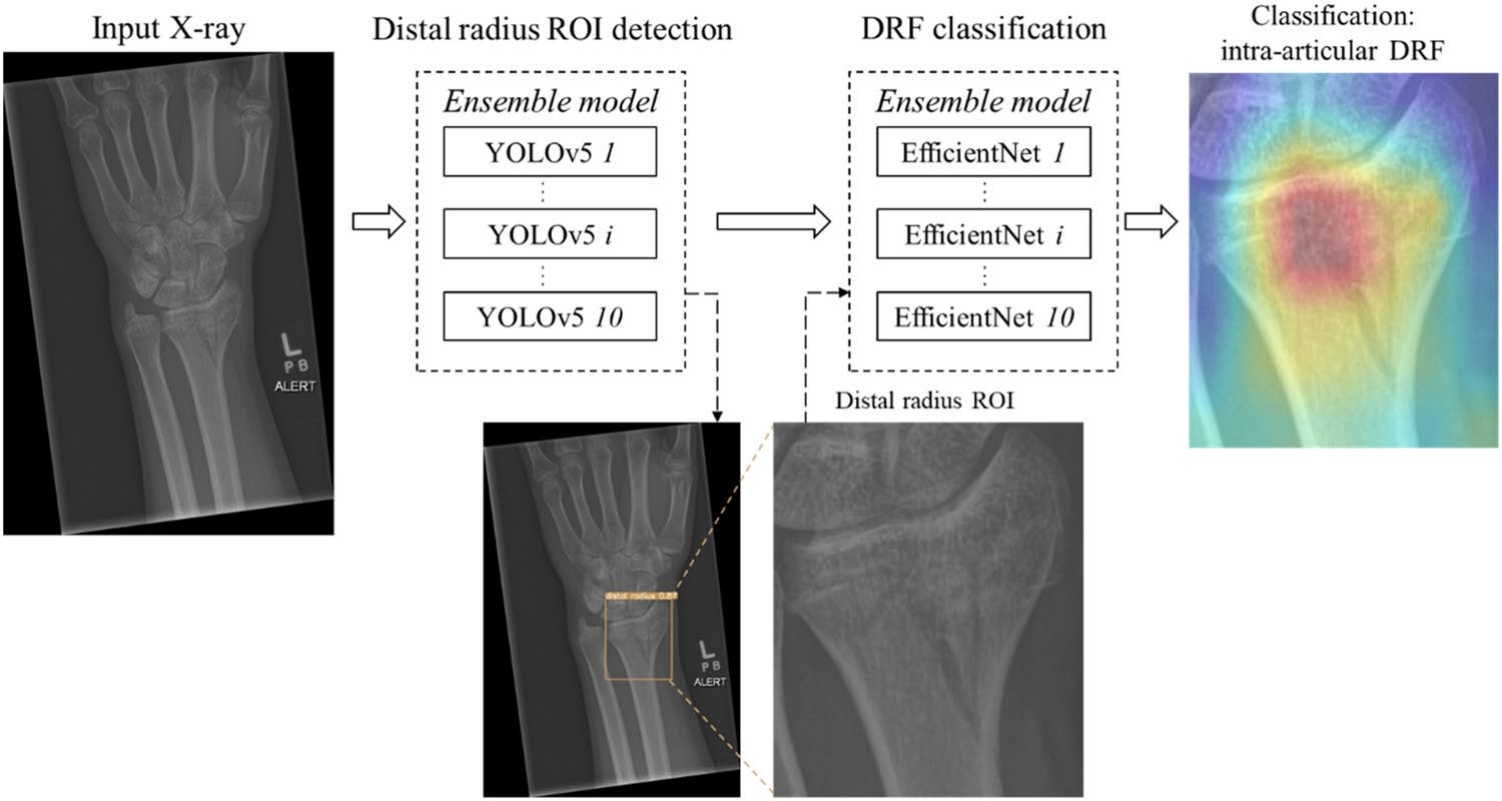
Diagram of the DRF classification framework

This study describes a two-stage DRF classification framework which firstly detects distal radius ROIs using an ensemble model of YOLOv5 networks and then classifies DRFs in the ROIs into intra- or extra-articular using an ensemble model of EfficientNet-B3 networks. The diagram of DRF class prediction is shown in Fig. 2.

#### Overview of YOLO and EfficientNet networks

The YOLO network [18] is one of the most effective real-time object detection algorithms. It only ‘looks’ at the entire image once by applying a single convolution network (Darknet [20]) on the whole image. YOLOv5 is a recently released YOLO architecture [14]. It introduced the cross stage partial network (CSPNet) [21] into the Darknet backbone to address the duplicate gradient problems, and adopted the path aggregation network (PANet) [22] as the neck for feature fusion to improve the information flow and localization accuracy [23]. During testing, YOLOv5 allows the ensemble of multiple base models by collecting all detections prior to filtering of predictions using non-maximum suppression (NMS) [24], which aims to improve generalization on unseen data. The YOLOv5s architecture, a small variant of YOLOv5 with approximately 7.3 M parameters, was adopted in this study.

EfficientNets describe a family of DL models that uniformly scale all dimensions (width, depth and resolution) of a baseline CNN using a compound coefficient to any target resource constraints [15, 25]. Starting from a baseline network EfficienNet-B0, if there are ${2}^{N}$ time more resources available, the network’s depth, width and image resolution can be expended by ${\alpha }^{N}$, ${\beta }^{N}$ and ${\gamma }^{N}$ respectively. $\alpha$, $\beta$ and $\gamma$ are constants decided by a small grid search on the original baseline model and selecting different $N$ generates EfficientNet-B1–B7. It has been demonstrated that EfficientNet could achieve better accuracy and efficiency compared with many existing CNN models (e.g. ResNet) which adopt arbitrary scaling practice [15]. In this study, the EfficientNet-B3 variant was adopted.

#### Experiment design

##### Network training

**Fig. 3:**
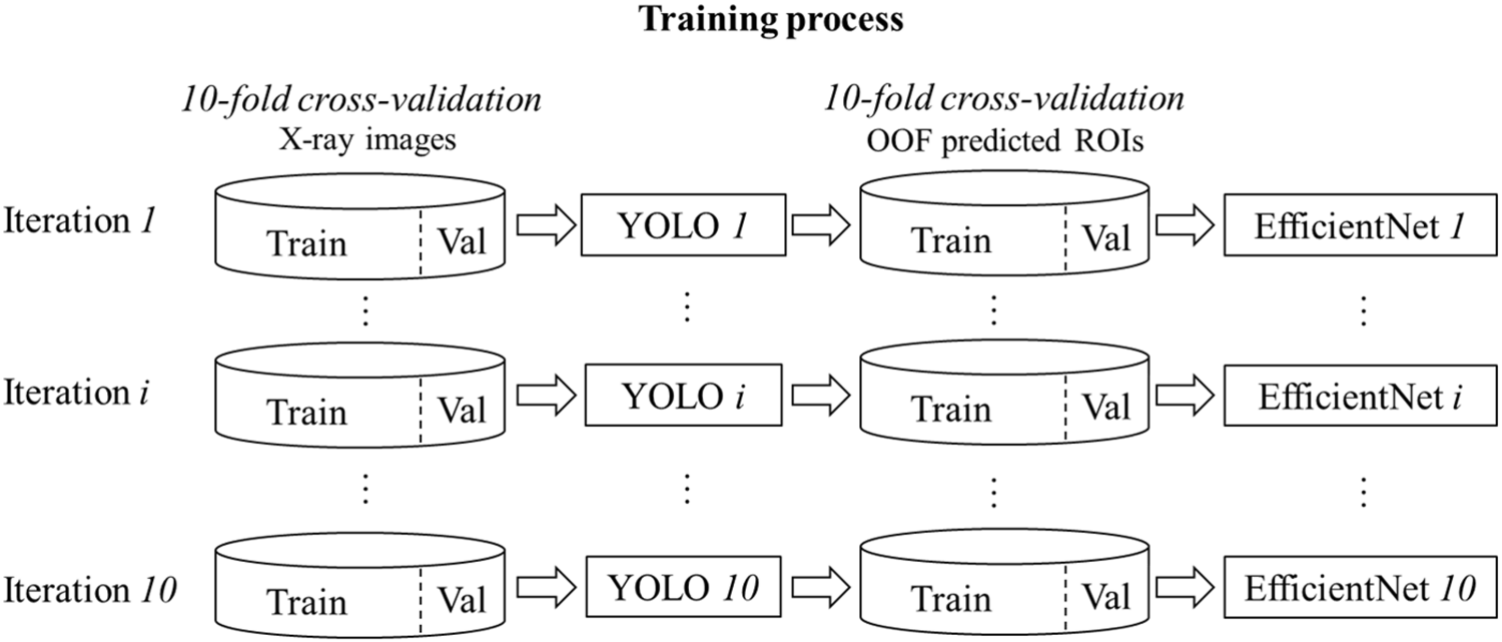
Training process for YOLO and EfficientNet. The YOLO network was trained within a tenfold cross validation, resulting in 10 YOLO base models YOLO *1*–*10*. These models were then applied on the validation sets to generate out-of-fold (OOF) ROI predictions. The EfficientNet was trained with a tenfold cross validation on the OOF predicted ROIs, generating 10 EfficientNet models EfficientNet *1*–*10*

For distal radius ROI detection, the YOLOv5s network was trained within a tenfold cross validation, generating 10 base models YOLO *1*–*10* as shown in Fig. 3. In each iteration of the cross validation, the network was trained for 80 epochs with a batch size of 16 and image size of $1280\times 1280$. Stochastic gradient descent (SGD) was used as the optimizer. The initial learning rate was set as 0.01 with a momentum of 0.937 and weight decay of 0.0005. A one-cycle learning rate scheduler policy [26] was adopted with a maximum and minimum learning rate of 0.01 and $0.2\times 0.01$. Augmentations including translation, scaling, horizontal flip and the mosaic augmentation [27] were applied during training. In each iteration of cross validation, the model with the best performance metric on the validation set was selected as the base model for ensemble. Here, the performance metric is a weighted combination of precision, recall and mean average precision [14]. The base models were also applied on the validation images to generate out-of-fold (OOF) ROI predictions which were used for training the EfficientNet classifiers in the next stage as shown in Fig. 3. During validation prediction, the confidence score threshold for ROI detection was set as 0.1.

The EfficientNet-B3 was also trained within a tenfold cross validation, resulting in 10 base models EfficientNet *1*–*10* as shown in Fig. 3. In each iteration, the model pretrained with ImageNet [28] was trained for 50 epochs with a batch size of 16 on the OOF predicted distal radius ROIs. The Adam optimizer was used with a learning rate of 0.001 and a cosine annealing scheduler. The ROI images were resized to $224\times 224$ and normalized to the mean and standard deviation of ImageNet. Augmentations including horizontal flip and the RandAugment [29] strategy were adopted during training. The model with the best area under the ROC curve (AUC) on each validation set was selected as the base model for ensemble. In this work, the intra-articular fracture is regarded as the positive class while the extra-articular as the negative one. All experiments were carried out on a NVidia Tesla P100 GPU with 16 GB Memory.

##### DRF classification framework testing using ensemble models and operating point selection

To extract the distal radius ROIs on the testing images, the model ensembling feature of YOLOv5 was enabled to fuse the 10 YOLO base models’ predictions. The predicted ROIs were then fed into the 10 EfficientNet base models. The predicted probability for each testing instance was calculated by averaging the probabilities across all base models. If a fracture was present in more than one image, the predicted probability of this fracture was set as the average of the probabilities across all images.

**Fig. 4:**
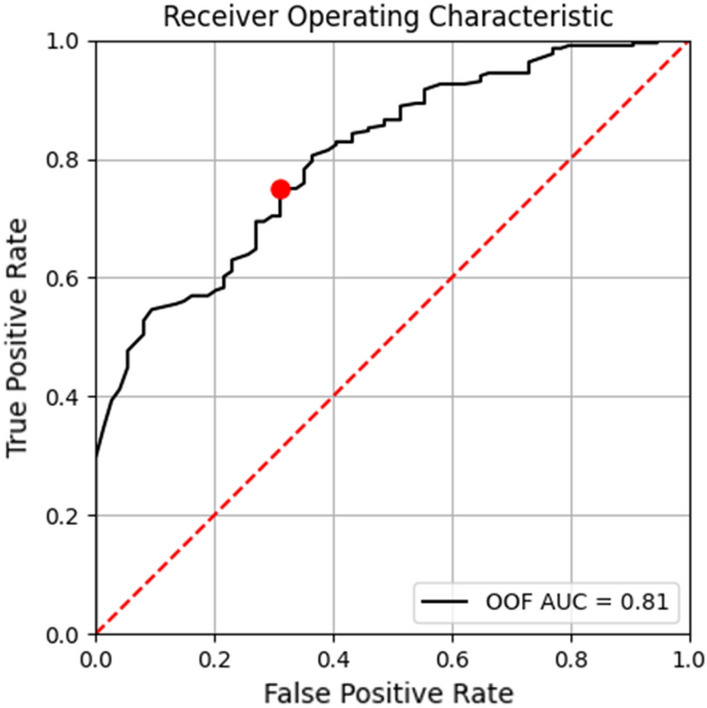
Out-of-fold prediction ROC on the validation data. The red dot marks the operating point selected based on the minimum distance approach

To perform the binary classification, a probability threshold $T$ was selected. A testing instance is classified as positive (intra-articular) if its predicted probability $P\ge T$, otherwise as negative (extra-articular). Given the class imbalance between the intra- and extra-articular fractures (ratio approx.3:1), the standard 0.5 probability threshold will bias towards the majority class, resulting in imbalanced true positive rate (TPR) and false positive rate (FPR). To address this issue, the minimum distance approach [16] was adopted to select the operating point and corresponding probability threshold $T$ based on the ROC curve of the OOF prediction on the validation data as shown in Fig. 4. On a ROC curve, the point (0, 1) has the highest TPR and lowest FPR. As depicted in Eq. 1, the minimum distance approach selects the point $j$ on the ROC curve which has the smallest distance to (0, 1) as the operating point. Based on this approach, the red dot on the OOF validation ROC in Fig. 4 was selected, where $T=0.79.$

1 $${argmin}_{j}\left(Distance \left(j\right)\right), Distance \left(j\right)= \sqrt{{(1-TPR(j))}^{2}+ {FPR(j)}^{2}}$$

## Results

### Distal radius ROI detection performance

**Fig. 5:**
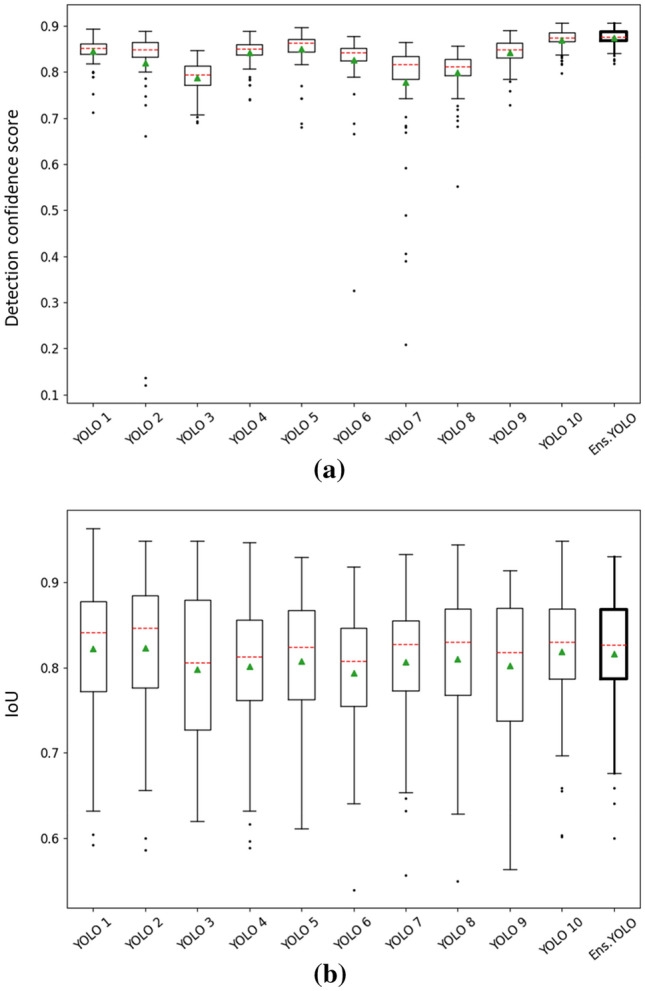
**a** The boxplot of detection confidence score of individual base models and the ensemble model on the test set. **b** The IoU boxplot of individual base models and the ensemble model on the test set. The red line stands for the median and the green triangle marks the mean of the elements. YOLO $i$ stands for the *i*th base model. Ens. YOLO stands for the ensemble model.

When applied on the unseen testing data to detect distal radius ROIs, the ensemble of YOLOv5 (YOLOv5s variant) networks achieved a TPR of 1 and a FPR of 0. Figure 5a illustrates the boxplot of detection confidence score of each base model and the ensemble model. The average confidence score generated by the ensemble model ($0.874\pm 0.018$) is higher than the best one achieved by the individual base models ($0.869\pm 0.023$, p-value = 0.0006 < 0.05). Figure 5b illustrates the boxplot of intersection over union (IoU) between the predicted ROI and the ground truth. The average IoU achieved by the ensemble model is $0.816\pm 0.071$, which is comparable to the highest one ($0.823\pm 0.084$, p-value = 0.39 > 0.05) yielded by individual base models.

Comparison experiments were also carried out using other YOLOVv5 variants, including YOLOv5m (medium) with approximately 21.4 M parameters and YOLOv5l (large) with approximately 47.0 M parameters. The YOLOv5m and YOLOv5l ensemble models achieved an average IoU of $0.804\pm 0.084$ (p-value = 0.06 > 0.05) and $0.821\pm 0.086$ (p-value = 0.43 > 0.05) respectively, which indicates that using larger YOLO variants did not result in significant improvement in distal radius ROI detection.

### Distal radius fracture classification performance

**Fig. 6:**
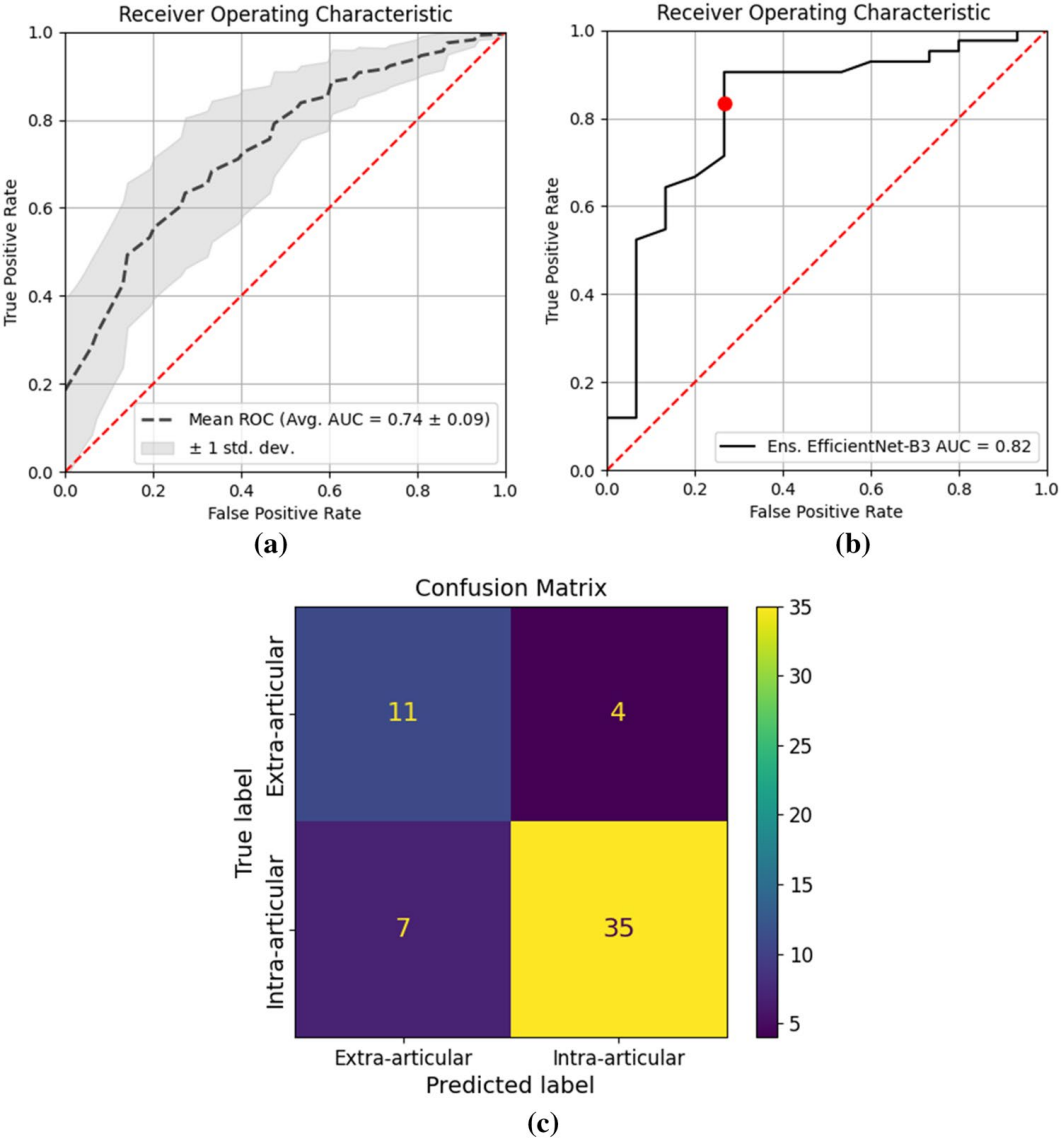
**a** The dash line represents the average ROC of all individual EfficientNet model predictions and the grey area illustrates the standard deviation of TPR at different FPR. **b** The ROC of the EfficientNet ensemble model, where the red dot marks the selected operating point based on the minimum distance approach. **c** confusion matrix

**Fig. 7:**
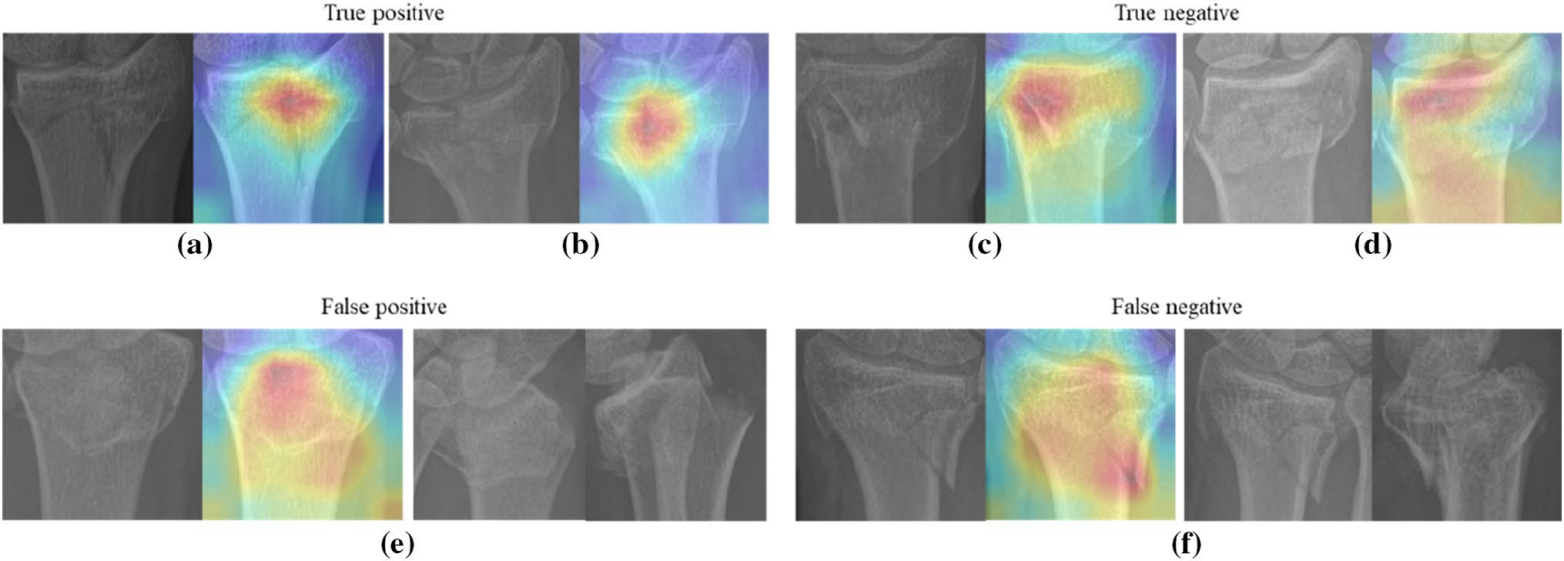
DRF classification examples with Grad-cam overlay on distal radius ROIs. **a** and **b** are true positive detections (intra-articular). **c** and **d** are true negative classifications (extra-articular). **e** and **f** are false positive and false negative classifications respectively. For the incorrectly classified examples (**e**) and (**f**), apart from the ROIs extracted from the PA view X-ray and the Grad-cam overlay (first two images for these cases), the relevant regions from the PA oblique and lateral views are also shown on the right side to provide additional information

The DRFs in the distal radius ROIs generated by the YOLO ensemble model were classified by the EfficientNet-B3 ensemble model as intra- or extra-articular fractures. Figure 6a illustrates the average ROC of the individual EfficientNet base models on the test set, while the grey area represents the standard deviation of TPR at different FPR across the ten base models. By fusing the predictions of all base models, the ensemble model yielded an AUC of 0.82 on the test set as shown in Fig. 6b. With the probability threshold selected based on the validation ROC using the minimum distance approach, the proposed framework achieved an accuracy of 0.81, TPR (sensitivity) of 0.83 and FPR (1-specificity) of 0.27 on the test set as marked by the red dot in Fig. 6b. The classification confusion matrix is shown in Fig. 6c. The whole framework takes approximately 2 s on average to classify each fracture. Examples of true positive (TP), true negative (TN), false positive (FP) and false negative (FN) classifications with Grad-cam [17] overlay can be found in Fig. 7, where the final Grad-cam heatmap is the average across all heatmaps from the ten individual models. It can be observed in Fig. 7 that the heatmaps can highlight the fractured area and the intersection between the fracture line and the articular surface. For the incorrectly classified DRFs, i.e., FP classification (e) and FN classification (f), the distal radius regions from the PA oblique and lateral views (not used in current framework) are also shown for a more comprehensive visualization of the fractures.

Table 2 shows the performance comparison between the proposed two-stage framework and several other approaches, including a one-stage framework which directly classifies the whole X-ray images using the EfficientNet-B3 ensemble model and two-stage frameworks which use YOLO ensemble model for ROI detection and ensemble classification model with other base networks (i.e. ResNet-18 [12], DenseNet-169 [30], Swin Transformer [31] and EfficientNet-B0, B1, B2 and B4 [15]). Evaluation metrics including AUC, accuracy, TPR, FPR, precision, recall, F1-score, and Cohen’s kappa score were calculated in Table 2. It can be observed that the proposed two-stage framework outperformed the one-stage approach in DRF classification. The EfficientNet family performed much better than ResNet-18, DenseNet169 and the Swin Transformer in the ensemble models. Compared with other EfficientNet variants, the EfficientNet-B3 ensemble model achieved higher accuracy, F1-sore and Cohen’s kappa score, indicating a more balanced ability to identify both classes and a stronger agreement to the ground truth annotation.

**Table 2:** Fracture classification performance comparison between the proposed two-stage method (in bold), a one-stage approach which directly classifies full X-ray images using EfficientNet-B3 ensemble model, and other two-stage frameworks using YOLO ensemble model for ROI detection and ensemble classification model with other base networks (ResNet-18, DenseNet169, Swin Transformer, EfficientNet-B0, B1, B2 and B4)

Networks used in the Ensemble model	AUC	Acc	TPR (Sen.)	FPR (1-Spe.)	Precision	Recall	F1-score	Cohen’s kappa score
*One-stage framework*								
EfficientNet-B3	0.69	0.68	0.86	0.80	0.75	0.86	0.80	0.07
*Two-stage framework*								
**YOLO + EfficientNet-B3 (proposed)**	**0.82**	**0.81**	**0.83**	**0.27**	**0.90**	**0.83**	**0.86**	**0.53**
YOLO + ResNet-18	0.42	0.26	0.10	0.27	0.50	0.10	0.16	− 0.10
YOLO + DenseNet169	0.50	0.28	0.02	0.00	1.0	0.02	0.05	0.01
YOLO + Swin Transformer	0.50	0.74	1.00	1.00	0.74	1.0	0.85	0.00
YOLO + EfficientNet-B0	0.80	0.72	0.71	0.27	0.88	0.71	0.79	0.38
YOLO + EfficientNet-B1	0.84	0.65	0.60	0.20	0.89	0.60	0.71	0.30
YOLO + EfficientNet-B2	0.83	0.79	0.83	0.33	0.88	0.83	0.85	0.48
YOLO + EfficientNet-B4	0.81	0.72	0.69	0.20	0.91	0.69	0.78	0.40

*Acc.* accuracy, *TPR* true positive rate (sensitivity), *FPR* false positive rate (1-specificity)

## Discussion

Extra-articular and intra-articular fractures are two major types of DRFs. Compared with extra-articular fractures, intra-articular DRFs affect the joint, which may require further evaluation and more complex treatment including surgical fixation. DRFs are challenging to identify due to the high variability and subtlety of fracture patterns. In this study, a two-stage DRF classification system is described which firstly uses the ensemble model of YOLO networks to localize the distal radius ROIs and then classifies the DRF into intra- and extra-articular fractures using the ensemble model of EfficientNet-B3. Evaluated on a clinical wrist X-ray dataset with high variability in imaging parameters and image quality, the proposed framework outperformed the single-stage approach which carries out classification directly on full X-ray images. The EfficientNet ensemble model also demonstrated better performance on DRF classification compared with other DL networks.

When examining a radiograph, clinicians often zoom in on the anatomic region of interest [32]. Based on this visual search pattern, the YOLOv5 ensemble model was adopted to localize the distal radius ROI, which detected all ROIs without false positives. Compared with the individual models generated from each iteration in the cross validation, the ensemble model showed more confidence in ROI detection and achieved a relatively high average IoU with smaller variance across testing images as shown in Fig. 5. This stage of ROI detection enabled the fracture classification stage to focus on the relevant anatomic region and remove redundant information/noise in the image, which allowed for better fracture classification performance in comparison to the single-stage workflow of directly using the whole image as shown in Table 2.

The second stage of the framework was developed to classify the DRFs in the distal radius ROIs as either intra- or extra-articular. The model ensemble strategy was also adopted to fuse all base models in this stage, as the classification performance of a single model varied substantially during testing as shown in Fig. 6a. It can be observed in Table 2 that the EfficientNet family outperformed other DL classification networks with EfficientNet-B3 obtaining the best overall performance. For large DL networks such as DenseNet169 and Swin Transformer, model fitting is difficult given the limited size and high variability of the data used in this study. Compared with the other networks, EfficientNet demonstrated greater effectiveness and robustness when learning from the limited data. Figure 7 illustrates several DRF classification examples, where the relevant regions such as the fractured area and the intersection between the fracture line and the articular surface were strongly activated on the Grad-cam heatmap. The proposed framework could correctly classify DRFs in Fig. 7a–d, however, failed to classify the DRFs in (e) and (f) solely based on PA views. It can be observed more clearly in the additional PA oblique and lateral views whether the fracture lines extent to the joint surface for the DRFs in Fig. 7e and f.

This work explores the feasibility of DRF classification using DL and has several recognised limitations due to the preliminary phase of study. First, the dataset used for training and evaluating the framework was relatively small. Acquiring a large size DRF X-ray dataset with reliable fracture class annotations is difficult since inter-observer variance often exists and the cases may require multiple clinician labels, which is laborious, time-consuming and resource intensive. For future work, more data will be acquired from collaborating hospitals to enlarge the dataset. Second, this study only used the PA view X-rays to establish the classification framework, however, clinicians normally combine information from all available views (PA, lateral and oblique if available) for clinical decision making. In the future, a multi-view network will be built incorporating both PA and lateral view images, which would aim to improve the classification performance.

## Conclusion

Identifying DRF types can provide useful information for further clinical evaluation and treatment planning. However, DRF classification is a challenging task due to the subtlety and complexity of fracture patterns. In this work, a two-stage ensemble DL framework for DRF classification was proposed. The described framework localizes distal radius ROIs using an ensemble model of YOLO networks and classifies the DRF into intra- and extra-articular fractures with the ensemble model of EfficientNet. Built and evaluated on a clinical wrist X-ray dataset that varies greatly in imaging parameters and image quality, the proposed DL framework achieved promising DRF classification performance with relevant fractured area highlighted using Grad-cam. Future research will expand the study to include a multi-view system with enriched data to assist clinicians in the evaluation of wrist fractures.
